# The Influence of Direct and Indirect Speech on Mental Representations

**DOI:** 10.1371/journal.pone.0065480

**Published:** 2013-06-12

**Authors:** Anita Eerland, Jan A. A. Engelen, Rolf A. Zwaan

**Affiliations:** 1 Department of Psychology, Open University, Heerlen, The Netherlands; 2 Department of Psychology, Erasmus University, Rotterdam, The Netherlands; Emory University, United States of America

## Abstract

Language can be viewed as a set of cues that modulate the comprehender’s thought processes. It is a very subtle instrument. For example, the literature suggests that people perceive direct speech (e.g., *Joanne said: ‘I went out for dinner last night’*) as more vivid and perceptually engaging than indirect speech (e.g., *Joanne said that she went out for dinner last night*). But how is this alleged vividness evident in comprehenders’ mental representations? We sought to address this question in a series of experiments. Our results do not support the idea that, compared to indirect speech, direct speech enhances the accessibility of information from the communicative or the referential situation during comprehension. Neither do our results support the idea that the hypothesized more vivid experience of direct speech is caused by a switch from the visual to the auditory modality. However, our results do show that direct speech leads to a stronger mental representation of the exact wording of a sentence than does indirect speech. These results show that language has a more subtle influence on memory representations than was previously suggested.

## Introduction

Suppose you are reading a story that contains the sentence *Joanne said: ‘I went out for dinner last night’*. Would it have made a difference if you had read the very similar *Joanne said that she went out for dinner last night* instead? Would it have made a difference, in other words, if the writer had used indirect speech rather than direct speech? The fact that the two different forms exist suggests that they serve different functions in linguistic communication. But what are these functions?

Indirect speech (e.g., *Joanne said that she went out for dinner last night*) is thought to be description-like, whereas direct speech (e.g., *Joanne said: ‘I went out for dinner last night’)* is considered to be more depiction-like [1]. We might construe this to mean that indirect speech focuses on what is said (the gist of a particular message) whereas direct speech focuses on creating a mental representation of the described situation. In terms of the Van Dijk and Kintsch [2] levels of representation, direct speech focuses on the surface structure whereas indirect speech focuses on the situation model. This distinction might be responsible for the fact that people perceive direct speech as more vivid and perceptually engaging than indirect speech [3], [4], [5]. Little is known about the effects of direct and indirect speech on the nature of mental representations that are formed during reading but research on this topic is emerging [3], [4], [6].

There is a great deal of evidence that people form mental representations of the described situation during language processing (e.g., [2], [7], [8], [9]). These representations are known as mental models or situation models. Subtly different linguistic constructions can have different effects on situation models. For example, various studies have examined the effects of grammatical aspect (e.g., [10], [11]) and negation [12] on the construction of situation models. What are the effects of using direct vs. indirect speech?

Recent studies are supportive of the idea that direct speech is more engaging than indirect speech. In one study, participants read short stories containing a direct or an indirect speech quotation. Context was manipulated so that either a fast or a slow speaking protagonist was implied. Reading times for direct speech were influenced by how fast the speaker spoke but reading times for indirect speech were not [5]. In an attempt to extend this finding, a recent study [6] explored whether not only speech rate but also the speed of the character’s movement influences reading times for direct and indirect speech quotations. People spent less time reading direct speech quotations when these utterances were described as being made quickly than as being made slowly. There was no effect of indirect speech quotations on reading times. There also was no effect for speed of movement on reading times. It thus seems that the use of direct speech causes the speaker’s voice to be more activated in the reader’s mind than the use of indirect speech. What we do not know is whether this more engaging experience influences our mental representations of described situations.

Given that direct speech is apparently perceived as more vivid than indirect speech, it seems likely that there are (subtle) differences in the mental representation of a given situation depending on whether this situation was described in direct or indirect speech. For example, objects that are present in the referential situation (i.e., the situation that is talked about) might be more accessible when they are talked about in direct speech than in indirect speech. This hypothesis is consistent with recent findings [5], [6] that readers are more likely to engage in perceptual simulations of a situation related in direct speech than in indirect speech. On the other hand, if, as Clark and Gerrig [1] suggest, indirect speech is more descriptive than direct speech, then we might expect situational information to be more strongly represented in indirect than in direct speech. We investigated this idea in Experiment 1.

In all of the experiments described in this paper, we used the same participant-recruitment and participant-exclusion plan, which is very similar to that of Zwaan and Pecher [13]. Criteria were set after we conducted the first experiment. For every experiment, except for Experiment 1a, we recruited 200 participants online through Amazon’s Mechanical Turk (http://www.mturk.com). All experiments were presented online in the Qualtrics survey research suite (http://www.qualtrics.com). Because we were interested in running native speakers of English only, we excluded participants who indicated at the end of the experiment to be no native speaker of English. We also excluded data from participants with low accuracy scores (<.75 in Experiment 2, <.80 in all other experiments). As these exclusion procedures often left us with unequal number of participants per counterbalancing list, we excluded data from the last-run participants of the longer list to create equal-length lists. After each experiment we asked participants 1) to guess what the purpose of the study was, 2) in what kind of environment they performed the experiment (regarding the amount of distraction and level of noise; on a 9-point scale), 3) what type of monitor participants used to perform the task, and 4) some demographical questions (age, gender, level of education, native language).

For all experiments, response times <300 ms and >10000 ms were removed, as they indicate extremely fast or slow responses. The remaining data were analyzed. Because standard significance testing might lead to false positives in large samples [14], [15], [16], we also calculated the posterior probability favoring the alternative hypothesis using the JZS Bayes Factor (*BF_01_*, calculated with Rouder's web based application at http://pcl.missouri.edu/bayesfactor), which provides the odds ratio for the null/alternative hypotheses given the data. A Bayes Factor of 1 means that they are equally likely, larger values (>3) indicate more evidence for the null hypothesis, and smaller values (<.33) indicate more evidence for the alternative hypothesis. Item analyses for Experiments 1–4 are reported in Appendix S1.

### Ethics Statement

The participants in all experiments were recruited online and voluntarily subscribed for participation in the described experiments. We did not obtain written consent. We did consult with the Ethics Committee of Psychology (ECP) at the Erasmus University Rotterdam, the Netherlands and receive a formal written waiver because the experiment was noninvasive and the results were analyzed anonymously.

## Methods

### Experiment 1a

In this first experiment, we investigated the accessibility of information regarding the referential situation that was either in direct or indirect speech. We used a probe recognition task to do so. Probe recognition tasks are commonly used to probe the strength of situational dimensions such as space [17], time [18], character goals [19], [20], and combinations thereof [21]. In a probe-recognition task, words are presented after a sentence. The participants’ task is to indicate as quickly as possible whether the word has occurred in the sentence they just read. Responses are usually very accurate but differences in response speed are thought to reflect differences in the strength with which situational information is active in the reader’s working memory [9]. For example, responses are faster when the probe word refers to an event that is still ongoing in the described situation than when the word refers to a past event [18]. Responses are also faster when the probe word refers to an object that is present in the described situation than when it refers to an absent object [12].

If direct speech is indeed perceived as more vivid than indirect speech, one might hypothesize, based on the findings of Yao and colleagues [3], that information that was presented in direct speech is more accessible than information presented in indirect speech. If, on the other hand, indirect speech is perceived as more descriptive than direct speech, then one might predict the opposite pattern. Our hypothesis was based on the idea that direct and indirect speech differ in terms of vividness and therefore we predicted that people should respond faster to probe words that were mentioned in direct speech than to probe words that were mentioned in indirect speech.

#### Participants

One hundred and eighty participants were recruited online of which 179 completed the experiment. The sample had a mean age of 34 (range = 18–75, 108 females). All participants were residents of the USA and received $1 for their participation, which required approximately 26 minutes. Ten participants did report another language than English as their native language. With the exclusion of these participants, our sample included 169 native speakers of English.

#### Materials and procedure

Participants first performed a lexical decision task in which they were randomly presented with eight strings of letters (one at a time). They had to indicate as fast as possible whether a given string was a word (m-key) or not (c-key). Four words and four non-words were included in this task. The lexical decision task was added to the actual experiment to familiarize participants with the task of making speeded responses to visual stimuli.

Next, participants read 48 short stories (24 experimental, 24 filler; adapted from [3] (see Appendix S2) online, sentence by sentence. Each story consisted of three sentences with the last sentence always being a direct or indirect speech quotation (see example story below). Two versions were created that differed regarding the last sentence of the experimental stories. Whenever the last sentence contained a direct speech quotation in one version, the sentence contained an indirect speech quotation in the other version. In both versions, half of the quotations were direct. This was true for both experimental and filler stories. All stories were presented in a random order.

### Example Story (*probe*)

It was 5.30 p.m. and everybody was ready to leave the office.

At one desk, Elaine was having a quick chat with Steven about her work.

**Direct:** She said: “The amount of *paperwork* is killing me at the moment. I feel completely exhausted.”

**Indirect:** She said that the amount of *paperwork* was killing her at the moment, and that she felt completely exhausted.

Participants performed a probe-recognition task directly after each story to test the accessibility of text information regarding the referential situation. Crucially, for the experimental stories the probe was always a noun that was mentioned in direct or indirect speech, so each experimental story required a ‘yes’ response. The probes that followed the filler stories were also nouns but they were never mentioned previously and thus required a ‘no’ response. All probes we used were never mentioned in one of the other stories (in case of the experimental stories, probes were only mentioned once). Response times to the probes were measured. To make sure participants read all stories properly, comprehension questions followed after 25% of the stories. The right answer to these questions was ‘yes’ 50% of the time. Three practice trials were included before the actual experiment started.

Each trial started with the first sentence of a story. Participants pressed the space bar when they had read a sentence to make the next sentence appear. Whenever participants pressed the space bar after the third sentence of the story, a fixation cross appeared in the middle of the screen for 1000 ms, followed immediately by the probe. Participants had to indicate as fast as possible whether this probe was mentioned in the story they just read (m-key) or not (c-key).

#### Results

We excluded data from participants with an accuracy <80% on the probes (eight participants) and data from one participant due to problems with the recording of response times. Finally, we excluded data from six last-run participants on one of the lists to make both lists equal regarding the number of participants. Data from the remaining 154 participants were analyzed. Unfortunately, there was a counterbalancing error involving one of the stories (it appeared in the same condition twice), so we excluded this item for all participants.

Mean response times to the probes are displayed in Table 1. In contrast to what we expected, there was no effect of speech, *t*(153) = 1.45, *p* = .15, *BF_01_* = 5.55. Accuracy levels were high (.96 for direct and.95 for indirect speech) and did not differ between conditions, |*t*| <1.

**Table 1:** Critical Dependent Measures from all Experiments.

	*N*	Direct M (SD)	Indirect M (SD)	*p*	Effect size (Cohen’s *d*)	*BF_01_* *
**Noun**						
Probe RT* 1a	154	1260 (420)	1225 (386)	.15	.09	5.55
Probe RT 1b	184	1357 (526)	1355 (495)	.93	.004	17.02
1a and 1b combined	338					12.79
Probe Accuracy 1a	154	.96 (.06)	.95 (.07)	.80	.15	15.17
Probe Accuracy 1b	184	.95 (.06)	.94 (.08)	.03	.14	1.66
1a and 1b combined	338					.002
**Adverb**						
Probe RT 2a	168	1209 (467)	1144 (446)	.001	.14	.03
Probe RT 2b	176	1210 (446)	1168 (420)	.03	.09	1.56
2a and 2b combined	344					.01
Probe Accuracy 2a	168	.93 (.10)	.94 (.08)	.1	−.11	4.27
Probe Accuracy 2b	176	.91 (.12)	.93 (.11)	.002	−.17	.16
2a and 2b combined	344					.06
**Adverb Additional sentence**						
Reading times 3a	172	2842 (986)	2916 (983)	.069	.08	3
Reading times 3b	174	3129 (1047)	3096 (1082)	.401	.03	12
3a and 3b combined	346					14
Probe RT 3a	172	1540 (602)	1537 (622)	.922	.00	16
Probe RT 3b	174	1613 (769)	1557 (709)	.086	.07	4
3a and 3b combined	346					8
Probe Accuracy 3a	172	.79 (.18)	.85 (.15)	.000	.36	.00
Probe Accuracy 3b	174	.82 (18)	.86 (.15)	.000	.32	.01
3a and 3b combined	346					.00
**Auditory Probe**						
Probe RT 4a	140	2607 (870)	2569 (890)	.28	.04	8.56
Probe RT 4b	144	3060 (1042)	2997 (1034)	.02	.06	1.20
4a and 4b combined	284					1.63
Probe Accuracy 4a	140	.95 (.07)	.94 (.07)	.54	.14	12.40
Probe Accuracy 4b	144	.92 (.13)	.93 (.10)	.20	−.09	6.74
4a and 4b combined	284					16.95
**Surface structure**						
*d’* *5a	188	1.88 (1.37)	1.55 (1.30)	.006	.24	.42
*d’* * 5b	188	1.90 (1.27)	1.57 (1.25)	.002	.26	.15
5a and 5b combined	376					.01
C* 5a	188	0.27 (0.79)	0.29 (0.64)	.96	−0.03	17.25
C 5b	188	−0.21 (0.67)	−0.19 (0.66)	.56	−0.03	14.62

* *BF_01_* = Bayes factor; RT = response time in milliseconds; *d’* = measure of sensitivity, C = measure of tendency to respond ‘yes’.

Because we did not determine all exclusion criteria before collecting the data, this study must be considered exploratory in nature. In Experiment 1b we tried to replicate our findings using the exact same settings as in Experiment 1a. Therefore, the study described as Experiment 1b is confirmatory rather than exploratory [22]. We followed this procedure for all experiments (see also [13]).

### Experiment 1b

#### Participants

Given that many psychology studies are underpowered [23] we started this experiment by conducting a power analysis with the program G*Power [24] to estimate the sample size needed to detect an effect of speech on accessibility of text information regarding the referential situation. According to this power analysis, at least 174 participants were needed to obtain statistical power at the recommended.80 level [25] (An anonymous reviewer suggested to use an ANOVA with repeated measures to estimate the sample size rather than a *t*-test. The F-test takes into account the real correlation between measures (.73, based upon the results of Experiment 1a) rather than an estimated correlation (.5). According to this alternative power analysis, at least 154 participants were needed to obtain statistical power at.80 level. The number of measures per condition was 12. The effect size was.10 based on the results of Experiment 1a). Because we anticipated that the sample would include non-native speakers of English, we recruited 216 participants online of which 209 completed the experiment. The sample had a mean age of 34 (range = 18–70, 117 females). All participants were residents of the USA and received $1 for their participation, which required approximately 28 minutes. We excluded the data from six participants because they reported another language than English as their native language. With the exclusion of these participants, our sample included 203 native speakers of English.

#### Materials and procedure

Except for the fact that we repaired the counterbalancing error of one of the experimental stories, the materials and procedure for this experiment were exactly the same as in Experiment 1a.

#### Results and Discussion

We excluded data from participants that had accuracy scores <.80 (11 participants). Data from one participant were excluded because he or she also participated in Experiment 1a and data from seven participants were removed to equalize both lists regarding the number of participants. Data from the remaining 184 participants were analyzed.

Mean response times to the probes are displayed in Table 1. Again, we found no effect of speech, *t*(183) = 0.09, *p* = .92, *BF_01_* = 17.02. Accuracy levels were somewhat lower than in Experiment 1a (.95 for direct and.94 for indirect speech) and there was a significant difference between conditions, *t*(183) = 2.18, *p* = .03, *BF_01_* = 1.66. So people responded slightly less accurately to probe words mentioned in indirect than in direct speech.

The results of Experiments 1a and 1b are similar and show reliable effects. In both studies, text information regarding a referential situation is not more accessible when this information was presented in indirect as compared to direct speech. Although this is not what we expected, Bayesian analyses indicated that the combined data of both experiments provide strong evidence for this null effect, *BF_01_* = 12.79.

Our results do not support our hypothesis that information regarding the referential situation is more accessible when this information was mentioned in direct as compared to indirect speech. But perhaps direct speech does not focus attention on the referential situation but rather on the communicative situation itself (i.e., the situation in which a conversation takes place). Evidence for this idea comes from a recent study by Stites and colleagues [6]. They found that people tend to read direct speech quotations faster whenever the talker speaks quickly (i.e., when someone was in a hurry) compared to when he was talking slowly. Conversely, they found no effect of talking speed on reading times on indirect speech quotations. It thus seems that the manner of speaking is more important in direct than in indirect speech. If this is true, then information about the manner of speaking should be more available after direct than after indirect speech. We investigated this idea in Experiment 2.

### Experiment 2a

#### Participants

Two hundred participants were recruited online of which 188 completed the experiment. The sample had a mean age of 32 (range = 18–66, 116 females). All participants were residents of the USA and received $0.75 for their participation, which required approximately 20 minutes. Eight participants reported another language than English as their native language. With the exclusion of these participants, our sample included 180 native speakers of English.

#### Materials and procedure

Participants first performed a lexical decision task (see Experiment 1a). Next, they read 48 sentences online (24 experimental sentences that were adapted from [6]; 24 fillers that we created ourselves; see Appendix S3). Each sentence consisted of a direct or an indirect speech quotation. Critically, an adverb was included in all sentences to provide information about the way of speaking. In the study by Stites and colleagues [6] only speed of speaking was manipulated. We decided to also use other kinds of adverbs (e.g., repeatedly, rudely, respectfully) so that testing the communicative situation was not limited to talking speed. We created two versions of the experiment that differed regarding the quotation in the sentence. Whenever the quotation was in direct speech in one version, it was in indirect speech in the other version. In both versions, half of the quotations were direct, whereas the speech quotation was indirect for the other half of the sentences. This was true for both experimental and filler items. All sentences were presented in a random order.

Participants performed a probe-recognition task directly after each sentence to test the accessibility of text information regarding the communicative situation. This time, for the experimental sentences the probe was always an adverb related to the way of speaking of the agent. As in our previous experiments, each experimental sentence required a ‘yes’ response. The probes that followed the filler stories were also adverbs but were never mentioned previously and thus required a ‘no’ response. Response times to the probes were measured. To make sure participants read all stories properly, comprehension questions followed after 25% of the stories. The right answer to these questions was ‘yes’ 50% of the time. Five practice trials were included before the actual experiment started.

Each trial started with the appearance of a sentence. Participants pressed the space bar whenever they had read a sentence to make the next one appear. After the third sentence, a fixation cross appeared in the middle of the screen for 1000 ms, followed immediately by the probe. Participants had to indicate as fast as possible whether this probe was mentioned in the sentence they had just read (m-key) or not (c-key).

#### Results

We excluded data from two participants for whom timing data somehow were not recorded and from participants with accuracy scores <75% (eight participants). The removal of these ten participants yielded unequal numbers of participants across lists. Data from the last-run participants of the longest list were removed so that both list were equal regarding the number of participants. Our analysis included data from the remaining 168 participants.

Mean response times to the probes are displayed in Table 1. We found a small but reliable effect. As in Experiments 1a and 1b, people were faster to respond to probes after reading an indirect than a direct speech quotation, *t*(167) = 3.51, *p* = .0006, *BF_01_* = .03. Accuracy levels were high (.93 for direct and.94 for indirect speech) and did not differ between conditions, |*t*|<1.

### Experiment 2b

#### Participants

Two hundred participants were recruited online and all completed the experiment. The sample had a mean age of 34 (range = 19–69, 115 females). All participants were residents of the USA and received $0.75 for their participation, which required approximately 20 minutes. Ten participants did not report English as their native language. With the exclusion of these participants, our sample included 190 native speakers of English.

#### Materials and procedure

The materials and procedure for this experiment were exactly the same as in Experiment 2a.

#### Results and Discussion

We excluded data from participants that had accuracy scores <.75 (nine participants). Data from five participants were removed to equalize both lists with respect to the number of participants. The remaining data (176 participants) were analyzed.

Mean response times to the probes are displayed in Table 1. We found a small effect showing that people respond faster to probes regarding the communicative situation after indirect than after direct speech quotations, *t*(175) = 2.20, *p* = .03, *BF_01_* = 1.56. Bayesian analysis shows that the evidence in favor of the alternative hypothesis must be considered ambiguous. Accuracy levels were high (.91 for direct and.93 for indirect speech) and differed between conditions, *t*(175) = 3.09, *p = *.002, *BF_01_* = .16.

The results of Experiment 2a show that text information regarding a communicative situation is more accessible when this information was presented in indirect as compared to direct speech. The results of Experiment 2b are ambiguous concerning the influence of speech on accessibility of information in respect of the communicative situation. However, Bayesian analysis indicates that the combined data of both experiments provide strong evidence for the conclusion that information regarding a communicative situation is more accessible after indirect than direct speech, *BF_01_* = .01. The alternative hypothesis (faster responses after indirect than direct speech) is a hundred times more likely, based on these data, than the null hypothesis.

We expected direct speech to make readers focus more on the communicative situation (i.e., the way of speaking) as opposed to the referential situation (i.e., the content of the speech) than indirect speech. However, our results do not support this hypothesis. In fact, we found that text information regarding the communicative situation is more accessible in indirect than in direct speech.

### Experiment 3a

In this experiment we wanted to examine an alternative explanation for our finding that text information regarding the communicative situation is more accessible in indirect than in direct speech. Perhaps direct speech is so engaging that it is more difficult to switch from the comprehension task to the probe recognition task after direct than indirect speech. To test this idea, we added a sentence that did not convey speech to each of our stimulus texts, such that the probe word was not presented immediately after the direct/indirect speech manipulation but after an intervening sentence.

If the probe-response advantage of indirect of over direct speech persists, then we can rule out that this is due to a larger task-switching effect in the direct speech condition. Moreover, by measuring reading times on the added sentence, we could examine whether switching from direct speech to non-speech incurs processing costs. If this is not the case, then this would provide supportive evidence that the probe-response disadvantage for direct speech found in Experiment 2 is not due to task switching.

#### Participants

Two hundred participants were recruited online of which 185 completed the experiment. The sample had a mean age of 34 (range = 18–69, 117 females). All participants were residents of the USA and received $1 for their participation, which required approximately 25 minutes. There were seven participants that did not report English as their native language. With the exclusion of these participants, our sample included 178 native speakers of English.

#### Materials and procedure

In this experiment, we added a last sentence to the sentences that were used in Experiment 2 after which the probe appeared. This sentence never contained direct or indirect speech (see Appendix S3). The procedure was the same as in Experiment 2, only this time we were also interested in reading times for all last sentences.

#### Results

Because removal of the data from nonnative speakers of English yielded unequal numbers of participants across lists, we removed the data from six last-run participants of the longest list. Data from the remaining 172 participants were analyzed.

Mean reading times for the last sentences and mean response times to the probes are displayed in Table 1. We found no effect of speech (direct vs. indirect) on reading times, *t*(171) = 1.83, *p* = .069, *BF_01_* = 3.18. There was also no effect of speech on response times to the probes, *t*(171) = 0.10, *p* = .92, *BF_01_* = 16.45. Accuracy levels were lower than in all previous experiments (.79 for direct and.85 for indirect speech). Importantly, however, we found a significant difference between conditions regarding accuracy scores, *t*(171) = 5.13, p<0.000001, *BF_01_* = .00009.

### Experiment 3b

#### Participants

Two hundred participants were recruited online of which 183 completed the experiment. The sample had a mean age of 33 (range = 18–66, 112 females). All participants were residents of the USA and received $1 for their participation, which required approximately 26 minutes. There were four participants that did not report English as their native language. With the exclusion of these participants, our sample included 179 native speakers of English.

#### Materials and procedure

The materials and procedure for this experiment were exactly the same as in Experiment 3a.

#### Results and Discussion

Because removal of the data from participants who were no native speaker of English yielded unequal numbers of participants across lists, we removed the data from five last-run participants of the longest list. Data from the remaining 174 participants were analyzed.

Mean reading times for the last sentences and mean response times to the probes are displayed in Table 1. As is Experiment 3a, we found no effect of speech (direct vs. indirect) on reading times, *t*(173) = 0.84, *p* = .401, *BF_01_* = 11.71. Also, the analysis regarding the response times to the probes yielded the same results as in Experiment 3a. There was no effect of speech on response times, *t*(173) = 1.73, *p* = .086, *BF_01_* = 3.80. Accuracy levels were comparable to those in Experiment 3a (.82 for direct and.86 for indirect speech) and differed again between conditions, *t*(173) = 3.96, *p* = .0001, *BF_01_* = .01.

In both experiments we found no effect of speech on reading times or response times. Moreover, the Bayesian analysis of the combined data provided very strong evidence for the null hypothesis regarding reading times (*BF_01_s* = 14) and response times to the probes (*BF_01_s* = 8). If it were more difficult to switch to a situation with no speech (e.g., the probe recognition task or a sentence that does not contain any speech) from direct speech than from indirect speech, one would expect differences in reading times for the last sentences. Given that we did not find such a difference, it seems unlikely that the results of Experiments 1 and 2 can be explained by more difficulty in switching to the probe recognition task after direct than after indirect speech.

Accuracy levels in Experiments 3a and 3b were lower than in our previous experiments. This finding can be explained by the fact that participants read another sentence before responding to the probe. In our previous experiments the probe immediately followed the sentence in which the probe was mentioned. This lower accuracy level may also explain why we did not find effects on probe-response times. There were fewer correct responses that could be entered into the analysis and participants may have emphasized accuracy over speed. This is why it is important that we found significant differences in probe accuracy between conditions. Participants were more accurate in responding to probes in the indirect than in the direct speech condition. Bayesian analysis of the combined data shows that the evidence is very strong for this conclusion (*BF_01_*<.001). This is in line with the results from Experiment 2, which suggest that indirect speech favors the communicative situation.

### Experiment 4a

So far, we have found no advantage (in terms of the accessibility of information during language processing) for direct speech over to indirect speech. It is possible that the ‘more vivid’ experience of direct speech does not necessarily influence information processing but prompts a switch from the visual modality (reading) to the auditory modality. In one recent study [3], participants read some short stories including a direct or indirect speech quotation while their brain activity was recorded. Participants showed more brain activity in the auditory cortex while reading direct as compared to indirect speech. This is consistent with the idea that silent readers are more likely to mentally simulate a character’s voice while reading to direct speech. Thus, if voice areas are more activated while reading direct as compared to indirect speech, then people should be primed to respond faster to auditory stimuli after reading direct speech than indirect speech. This idea is consistent with the modality switching effect (e.g., [26], [27], [28]). It also explains why direct-speech responses to visual probes were slower than expected in our previous experiments; participants had mentally shifted away from the visual modality.

To test this idea, we presented participants with spoken probe words rather than written ones (as in Experiments 1–3). Because of the just-described neuroimaging findings [3], we expected participants to respond faster to the probe after direct than after indirect speech because reading direct speech activates the auditory cortex more strongly than indirect speech.

#### Participants

Two hundred participants were recruited online of which 193 completed the experiment. The sample had a mean age of 35 (range = 18–67, 125 females). All participants were residents of the USA and received $1 for their participation, which required approximately 28 minutes. There were six participants that did not report English as their native language. With the exclusion of these participants, our sample included 187 native speakers of English.

#### Materials and procedure

Instead of the lexical decision task, we had participants perform a categorization task first. They were auditorily presented with four fruits (*grape*, *lemon*, strawberry, mango) and four animals (horse, *tiger*, *turtle*, rabbit; words in Italic were pronounced by a male). Participants had to decide as fast as possible whether the word they heard was a fruit (m-key) or an animal (c-key). Words were presented in random order. We included this task to familiarize participants with the task of making speeded responses to auditory stimuli. They were also instructed to use this task to set the volume of their computer to the right level.

Next, participants read the same 48 three-sentence stories that we used in Experiment 1 and performed a probe recognition task. However, this time, the probes were presented auditorily instead of visually. The pronounced words were collected from http://www.merriam-webster.com/. Some stories were slightly changed to make sure that whenever the probe was pronounced by a male it was also the case that a male spoke in the story (and not a female). We did so because we know that people encode features of speakers’ utterances, like gender [29], and we wanted to prevent mismatch effects. After each last sentence of a story, a fixation cross appeared on the screen for 1000 ms. Then participants heard an auditory probe and indicated as fast as possible whether the word they heard was present in the story they just read (m-key) or not (c-key).

To make sure participants read all stories properly, comprehension questions followed after 50% of the stories. The right answer to these questions was ‘yes’ 50% of the time. Three practice trials were included before the actual experiment started.

#### Results

We excluded data from participants with an accuracy <80% (38 participants). Furthermore, we excluded data from nine last-run participants on one of the lists to make both lists equal regarding the number of participants. The remaining data (140 participants) were analyzed.

Mean response times to the probes are displayed in Table 1. Although we expected people to respond faster to an auditory probe after reading direct as compared to indirect speech, we found no effect of speech on response times to the probes, *t*(139) = 1.08, *p* = .28, *BF_01_* = 8.56. Accuracy levels were high (.95 for direct and.94 for indirect speech) and did not differ between conditions, |*t|*<1.

### Experiment 4b

#### Participants

Two hundred participants were recruited online of which 189 completed the experiment. The sample had a mean age of 32 (range = 18–65, 116 females). All participants were residents of the USA and received $1 for their participation, which required approximately 30 minutes. There were eight participants that did not report English as their native language. With the exclusion of these participants, our sample included 181 native speakers of English.

#### Materials and procedure

The materials and procedure for this experiment were exactly the same as in Experiment 4a.

#### Results and Discussion

We excluded data from participants with an accuracy <80% (31 participants) and from six last-run participants on one of the lists to make both lists equal regarding the number of participants. The remaining data (144 participants) were analyzed.

Mean response times to the probes are displayed in Table 1. We found a very small effect. Although we expected people to respond faster to an auditory probe after reading direct as compared to indirect speech, we found an effect of speech on response times to the probes that was the opposite of this, *t*(143) = 2.28, *p* = .02, *BF_01_* = 1.2. Accuracy levels were high (.92 and.93) and did not differ between conditions, |*t|*<1.

The results of Experiment 4a show that there was no effect of speech on response times to auditorily presented probes, while the results of Experiment 4b show a very small effect favoring indirect speech. We thus ended up with mixed effects. Moreover, Bayesian analysis of the combined data provides no clear evidence for the null or the alternative hypothesis regarding response times (*BF_01_s* = 1.63).

The results of Experiment 4 do not support the idea that the more vivid experience of direct speech is caused by a switch from the visual to the auditory modality. We also tested the idea of auditory priming by direct speech in four other experiments (two exploratory and two confirmatory ones). In the first of these experiments, participants read the same 48 stories that we used in Experiments 1 and 4. However, after each last sentence, participants were presented with either a high (650 Hz) or a low (450 Hz) tone. They were instructed to decide as fast as possible whether the tone they heard was either high (650 Hz, always presented after the experimental items) or low (450 Hz, always presented after fillers). In another study, we replaced the tones by the spoken words ‘right’ and ‘left’. Participants decided as fast as possible whether the word they heard was either ‘right’ (experimental items) or ‘left’ (fillers). In none of these experiments we found an effect of speech on response times.

Our findings do not seem to be consistent with the literature. However, an experiment by Kurby, Magliano, and Rapp [30] on auditory imagery experiences (AIEs) during silent reading of direct speech yielded results similar to ours. In this study, participants first listened to dialogues between two characters. Then they read several texts, some of which they heard before, while others were new. While participants read those texts they performed a probe recognition task. Probes were auditorily presented and were either in the voice of the character that originally pronounced that word (match condition) or in the voice of the other character (mismatch condition). Participants were faster in the match than in the mismatch condition but this was only true for familiar scripts. In other words, people only had AIEs during silent reading of direct speech when they had previously experienced the same voice in the same situation. In our experiment, participants had prior experience with the voices that pronounced the probe words but not with the particular context in which they appeared. The fact that we did not find a priming effect of direct speech on auditory probes is therefore consistent with the results of Kurby and colleagues [30].

So far, we have found no evidence that direct speech enhances the availability of information about the referential and communicative situation relative to indirect speech. If anything, we have found (some) evidence to the contrary. However, so far we have only tested mental representations at the level of situation models (whether these are models of the referential or the communicative situation). It might be the case that the influence of direct speech takes place at another level of mental representation. According to van Dijk and Kintsch’s [2] classic model, linguistic input is represented at three levels: the surface structure (a representation of the exact wording of an utterance), the textbase (a representation of the explicitly stated meaning of an utterance), and the situation model (a representation of the referential situation). It is plausible that direct speech influences mental representations at the level of the surface structure. As we mentioned earlier, direct speech is thought to focus more on the exact words, whereas the gist of a particular message is the focus of indirect speech [1]. A recent study has reported initial evidence for this idea [31]. Participants were presented with a text. Then the text appeared again and participants were instructed to report any difference between the two texts. Speech was manipulated (direct vs. indirect) but also word-change. There could be no change at all between the two texts, there could be a semantically related word-change (flatmate – roommate), or a distantly related word-change (flatmate – brother). Change detection was significantly better in direct than in indirect speech. The authors therefore conclude that the exact wording of what was said by a story protagonist is critical for direct but not for indirect speech.

Based on these results, we expected people to focus more on the exact words in direct speech than in indirect speech. In Experiment 5 we tested this idea.

### Experiment 5a

#### Participants

Initially, we recruited 200 participants, but because of a large number of non-native speakers in two of our four lists, we decided to run a few more participants in these lists. In total, 214 participants were recruited online and all completed the experiment. The sample had a mean age of 34 (range = 15–66, 116 females). All participants were residents of the USA and received $0.5 for their participation, which required approximately 18 minutes. There were 15 participants that did not report English as their native language and one participant reported to be 15 years of age. With the exclusion of these participants, our sample included 198 adults who were native speakers of English.

#### Materials and procedure

Participants read all 24 experimental stories, sentence by sentence, that we used in Experiment 1. After each last sentence, a fixation cross appeared on the screen for 1000 ms. Then a sentence appeared and participants indicated whether this sentence was exactly the same as one of the sentences of the story they just read (m-key) or not (c-key). For half of the stories, the sentence that appeared after the fixation cross was exactly the same as the last sentence of the story (which was always a sentence in direct or indirect speech). For the other 12 stories, the sentence that appeared after the fixation cross was a paraphrase of the last sentence of the story (see example story below). We created four lists, so that we could manipulate speech (direct vs. indirect) and referential sentence (literally vs. paraphrase) within stories.

### Example Story (Paraphrase)

It was 5.30 p.m. and everybody was ready to leave the office.

At one desk, Elaine was having a quick chat with Steven about her work.

**Direct:** She said: “The amount of paperwork is killing me at the moment. I feel completely/totally exhausted.”

**Indirect:** She said that the amount of paperwork was killing her at the moment, and that she felt completely/totally exhausted.

To make sure participants understood that we were looking for subtle differences between sentences, we presented them with three practice trials. They received feedback on their responses during these trials.

#### Results

We excluded data from ten last-run participants on three of our four lists to make all lists equal regarding the number of participants. Data from the remaining 188 participants were analyzed.

We computed *d’* scores [32]. To be able to use *d’*, we converted scores of 1 and 0 to.99 and.01 respectively [33]. ‘Yes’ responses to literal statements were considered hits, whereas ‘yes’ responses to paraphrases were counted as false alarms. Mean *d’* scores by condition are displayed in Table 1. The results show a medium effect of speech on the ability to detect subtle changes in surface structure even though the textbase and situation model of the message remained the same, *t*(167) = 2.76, *p = *.006, *BF_01_* = .42. Participants were better at remembering the exact words that were used in direct than in indirect speech.

This difference cannot be explained by bias. We found no difference between conditions (direct vs. indirect speech) regarding the tendency to respond ‘yes’, |*t|*<1 (see C-scores in Table 1).

### Experiment 5b

#### Participants

Two hundred and one participants were recruited online (i.e., most likely due to technical issues, we ended up with data from 51 participants on one of the list) of which 200 completed the experiment. The sample had a mean age of 33 (range = 18–69, 124 females). All participants were residents of the USA and received $0.5 for their participation, which required approximately 18 minutes. There were eight participants that did not report English as their native language. With the exclusion of these participants, our sample included 192 native speakers of English.

#### Materials and procedure

The materials and procedure for this experiment were exactly the same as in Experiment 5a.

#### Results and Discussion

We removed data from four last-run participants to equal all four lists regarding the number of participants. The remaining data (188 participants) were analyzed.

Again, we computed *d’*scores (see Table 1) and we found a significant effect of speech on the ability to detect subtle changes in texts even though the gist of a message remained the same, *t*(187) = 3.14, *p* = .002, *BF_01_* = .15. So, also in this confirmatory experiment, participants remembered the exact words that were used better after direct than indirect speech. This effect is due to differences in sensitivity because we found no differences with respect to the tendency to respond ‘yes’ between conditions, |*t|*<1 (see C-scores in Table 1).

Although the effect we found was stronger for Experiment 5b than for Experiment 5a (due to smaller SDs in the last experiment), the results of both experiments are similar. Participants were better at remembering the exact words that were used, indicating a more prominent surface representation, after direct than after indirect speech. Bayesian analysis of the combined data also showed strong evidence in favor of the alternative hypothesis, *BF_01_s* = 0.01.

## General Discussion

Language can be viewed as a tool that “allows us to shape events in each other’s brains with exquisite precision” [34]. Ultimately, language comprehension amounts to creating a mental representation of the state of affairs described in an utterance. But how do subtle differences in the form of an utterance have their effect on how its contents are represented? In a series of experiments we sought to answer this question for direct and indirect speech quotations, which make up a major part of everyday communication. Our findings suggest that direct and indirect speech quotations influence mental representations at different levels.

Although direct speech is perceived as more vivid and is thought to be more engaging than indirect speech, we did not find support for the idea that direct speech makes textual information regarding the referential (Experiment 1) or the communicative situation (Experiment 2) more accessible. In fact, we observed no effect of speech in Experiment 1 and an advantage for indirect speech Experiment 2. We were able to rule out that this latter finding was due to greater task-switching costs after direct than after indirect speech (Experiment 3).

At first, these results seem puzzling but they can be explained by the distinction proposed by Clark and Gerrig [1]. According to these authors, indirect speech quotations are a descriptive form of language which means that they are aimed at conveying the gist of an utterance without necessarily drawing attention to its specific realization. Direct speech, on the other hand, is a depictive form of language. It offers the listener a more direct perceptual experience – comparable to looking at a Picasso painting itself, rather than reading a description of that painting. We explored whether this more direct perceptual experience – in this case of a person speaking – involved a switch from the visual to the auditory modality, as suggested by Yao and colleagues [3]. No evidence was found in support of this idea (Experiment 4). A possible explanation for this lack of support might be that the probe recognition task differs from the methods that were used in previous studies on direct and indirect speech and measured sentence processing [3], [4], [5], [6]. However, the absence of a priming effect of direct speech on auditory probes is consistent with the results showing that for auditory priming effects to occur, prior experience with a particular voice in the same context is required [30]. In our experiment, participants did have prior experience with the voices that pronounced the probe words but not with the particular contexts in which they occurred. How is it possible that people perceive direct speech as more vivid and engaging than indirect speech, and yet we found no clue that it makes the mental representation of the referential situation more accessible, or the depicted speech act more perception-like? Taking a cue from a well-known model of mental representations [2], we hypothesized that direct and indirect speech influence these representations at different levels (just as genre expectations have been found to do [35]). We found support for this idea. Participants showed superior memory for the exact wording of an utterance when it had the form of a direct speech quotation as compared to an indirect speech quotation (Experiment 5). Apparently, direct speech makes the exact wording of an utterance more salient, enhancing memory for the surface structure of the utterance, whereas indirect speech leads comprehenders to focus more on constructing a situation model.

To summarize, we have systematically addressed several potential consequences of the use of direct versus indirect speech quotations for comprehenders’ mental representations. As it turned out, not all experiments showed an effect in the expected direction or even an effect at all. Nevertheless, these results must be considered informative. Given the large numbers of participants, our experiments had sufficient statistical power to detect possible effects. Moreover, we used Bayesian analysis to determine the posterior probability of the null hypothesis and the alternative hypothesis for each experiment. This approach allows one to combine the results of multiple experiments to compute a single Bayes factor. By doing so with already large samples, we were able to put confidence in our claims regarding the null hypotheses, which would not be possible with the standard procedure of null hypothesis significance testing alone.

Although some of our results seem to be at odds with earlier findings in the literature, they need not be mutually exclusive. For instance, while the effect of implied talking speed on actual reading times may be a pervasive phenomenon, other aspects of the communicative situation [5], [6], such as a talker’s voice or manner of speaking, may only be simulated under specific conditions.

Together, our experiments paint a slightly complex, but coherent picture of the effect of direct and indirect speech quotations on comprehenders’ mental representations. While direct speech quotations make the exact wording of an utterance more memorable, this does not necessarily hold for the information it conveys.

## Supporting Information

Appendix S1 **Results of the item analyses for experiments 1–4.**
(DOC)

Appendix S2 **Materials that were used in Experiments 1, 4, and 5.**
(DOC)

Appendix S3 **Materials that were used in Experiments 2 and 3.**
(DOC)
