# Supplementary material for: The Influence of Direct and Indirect Speech on Mental Representations
Source: PLoS One. 2013 Jun 12;8(6):e65480. doi: 10.1371/journal.pone.0065480 (PMC3680483; doi:10.1371/journal.pone.0065480)
Supplement: Appendix S1 — Results of the item analyses for experiments 1–4. (DOC) [file pone.0065480.s001.doc]

Appendix S1 – Results of the item analyses for experiments 1 - 4

|  | *N* | *p* | *|t|* |
| --- | --- | --- | --- |
| **Noun** |  |  |  |
| Probe RT 1a | 23 |  |  |
| Probe RT 1b | 23 |  |  |
| **Adverb** |  |  |  |
| Probe RT 2a | 24 | .03 | 2.38 |
| Probe RT 2b | 24 | .07 | 1.90 |
| **Adverb**  **Additional sentence** |  |  |  |
| Reading times 3a | 24 |  | < 1 |
| Reading times 3b | 24 |  | < 1 |
| Probe RT 3a | 24 |  | < 1 |
| Probe RT 3b | 24 | .09 | 1.77 |
| Accuracy 3a | 24 | .00 | 5.93 |
| Accuracy 3b | 24 | .00 | 3.78 |
| **Auditory Probe** |  |  |  |
| Probe RT 4a | 24 |  | < 1 |
| Probe RT 4b | 24 |  | < 1 |
