# Supplementary material for: The Influence of Direct and Indirect Speech on Mental Representations
Source: PLoS One. 2013 Jun 12;8(6):e65480. doi: 10.1371/journal.pone.0065480 (PMC3680483; doi:10.1371/journal.pone.0065480)
Supplement: Appendix S2 — Materials that were used in Experiments 1, 4, and 5. (DOC) [file pone.0065480.s002.doc]

Appendix S2 - Materials that were used in Experiments 1, 4, and 5 (**D** = direct speech quotation, **I** = indirect speech quotation, **Q** = comprehension question*, probes used in Experiments 1 and 4 are underlined).

Experimental items

A Barnes & Nobles book store had recently opened in Seattle.

Today, Alexia, a young mother living nearby, came in and asked for advice.

**D:** She said: “It’s my son’s birthday tomorrow and I would like to buy him a storybook on adventure.”

**I:** She said that it was her son’s birthday soon and she would like to buy him a storybook on adventure.

**Q:** Did Alexia go to a book store in Seattle?

It was 5.30 p.m. and everybody was ready to leave the office.

At one desk, Elaine was having a quick chat with Steven about her work.

**D:**She said: “The amount of paperwork is killing me at the moment. I feel completely exhausted.”

**I:** She said that the amount of paperwork was killing her at the moment, and that she felt completely exhausted.

Derek’s birthday was just a couple of days away and his girlfriend Ruby had arranged something special.

Tonight Ruby decided to unveil the plan that she kept hidden from him for so long.

**D:** Over dinner, she said to Derek: “Well, your birthday is coming up soon, so I booked you a ticket to New York City to visit the opera.”

**I:** Over dinner, she revealed to Derek that since his birthday was coming up soon, she booked him a ticket to New York City to visit the opera.

**Q:** Was Ruby’s birthday coming up soon?

Alison and Nick travelled to Beijing during the Olympics.

Alison was amazed by the fine fabric for sale when they entered a big market.

**D:** She said to Nick: “I could buy all of it! The silk feels so incredibly smooth.”

**I:** She said to Nick that she could buy all of it, and that the silk felt so incredibly smooth.

In the office, Ned told Daniela that his car had been damaged during an accident the other day.

Daniela was sorry to hear that and tried to comfort him.

**D:** She said: “Well, at least you should get some money back from the insurance.”

**I:** She said that at least he should get some money back from the insurance.

**Q:** Had Ned’s car been damaged?

Jenny and Irvin were about to start a holiday trip to Barcelona.

When they arrived at the airport to check in, Jenny noticed that a heavy thunderstorm was brewing outside.

**D:** She complained: “That’s so unfair! I’m sure the plane will be delayed.”

**I:** She complained that this was so unfair because she was sure that the plane would be delayed.

Morton needed to speak to his project supervisor Marianne at her office.

When he asked whether she could spare a minute, she seemed rather busy.

**D:** She replied: “I’m really sorry, but I have to finish grading this paper first.”

**I:** She replied that she was really sorry and that she had to finish grading a paper first.

Marcus and Helen were planning a trip to Europe for their wedding anniversary.

Marcus was leaning more towards Prague while Helen preferred Rome.

**D:** Marcus explained: “Okay, Rome is a nice city, but the Czechs actually have much better beer.”

**I:** Marcus explained that Rome is a nice city, but that the Czechs would actually have much better beer.

Brian invited some of his mates home and started making a mess in the kitchen.

His mum was obviously not happy about this, and Brian was quick to offer an excuse.

**D:** He told her: “Well, we just had a couple of drinks because the pub is already closed.”

**I:** He told her that they just had a couple of drinks because the pub was already closed.

Jacob had promised Cindy to buy a bonsai tree to decorate their living room.

When Cindy wanted to place the plant by the television, he tried to persuade her of an alternative option.

**D:** He said: “Look, if you do that, there will be no sunlight for the bonsai, so it's better to place it by the window.”

**I:** He said that if she did that, there would be no sunlight for the bonsai, so it was better to place it by the window.

**Q:** Did Jacob want to place the plant by the television?

Luke and his friends were at the cinema.

Luke wasn’t particularly keen on romantic comedies, and he was complaining a lot after the film.

**D:** He said: “That movie was terrible! I’ve never been so bored in my life.”

**I:** He said that the movie was terrible and that he had never been so bored in his life.

During a coffee break, university teachers Robert and Isabel met in the common room.

Robert was slightly irritated with the mess in the kitchen.

**D:** He said to Isabel: “Look at this! Certain people must always leave their dirty mug on the table.”

**I:** He said to Isabel that, obviously, certain people must always leave their dirty mug on the table.

**Q:** Was Isabel complaining about the dirty mug?

It was a quarter past seven and the Korean restaurant was packed.

Debbie and Mike were very pleased with the meal and Debbie in particular was enthusiastic.

**D:** She said: ''We have to come here more often. The fish was really tasty and the soup was absolutely delicious.''

**I:** She said that they should go there more often because the fish was really fresh and the soup was absolutely delicious.

In order to finish the project in time, Audrey had been working in the office from 7 a.m. to 8 p.m. without taking a break.

Her colleague Sean just came back from dinner with a big smile on his face, which obviously upset her a bit.

**D:** She complained: ''It's so unfair! My stomach has been rumbling all day. I could eat a whole elephant.''

**I:** She complained that it was so unfair because her stomach had been rumbling all day, and that she could eat a whole elephant.

**Q:** Did Audrey take a break from work?

Cheryl and Barry were in the waiting lounge for their honeymoon flight to Paris.

Shortly before boarding time, Cheryl noticed that there was something wrong with her carry on luggage.

**D:** She shouted: '' There's a hole in my bag! Don't tell me I've lost my passport!...''

**I:** She shouted that there was a hole in her bag. She was worried that she had lost her passport.

**Q:** Was Cheryl’s bag broken?

Jessica and John were enjoying a hot and sunny day at the holiday resort in Florida.

When they were heading towards their usual spot on the beach, Jessica was slightly overwhelmed by the heat.

**D:** She said: ''Phew, this is almost a bit too hot for me! Perhaps we should first go back for a drink under the awning.''

**I:** She said that it was almost a bit too hot for her, and suggested to first go back for a drink under the awning.

In the small town of Oban lies this charming little pub.

One day, famous writer Aileen burst in with a big smile on her face.

**D:** She said: ''Guess what - my new novel has been accepted for publication! I'll buy everyone a round.''

**I:** She said that her new novel had been accepted for publication and that she wanted to buy everyone a round.

It was Sunday afternoon when housewife Heather was tidying up the bedroom.

After just two minutes, she came out of the bedroom and confronted her husband Ben.

**D:** She said: ''I can't believe how messy you are! The first thing I found on the floor was your dirty pyjamas!''

**I:** She said that she could not believe how messy he was, and that the first thing she found on the floor was his dirty pyjamas.

**Q:** Did Ben leave his pyjamas on the floor?

It was getting quite late and most of Andrea’s friends were about to leave her party.

Scott in particular seemed to have had a great time when he was leaving.

**D:** He said: ''Sorry about the crack in that glass. The party was absolutely fantastic.''

**I:** He apologized for the crack in that glass, adding that the party was absolutely fantastic.

**Q:** Did Scott throw a party?

It was 11 a.m. in the morning when the fire alarm went off.

Hearing people running down the hallways, Mary grabbed her jacket and burst into Peter’s office next door.

**D:** She shouted: ''Peter, we have to leave immediately because the building is on fire!''

**I:** She urged Peter to leave immediately because the building was on fire.

The West End festival was coming soon and the organizers were discussing details of the live television broadcast.

The current issue was where to set the television cameras and John was offering his plans.

**D:** He said: ''We might need television cameras along High Street, and possibly even a helicopter to cover the whole area.''

**I:** He suggested that they might need television cameras along High Street and possibly even a helicopter to cover the whole area.

**Q:** Was the West End festival coming up?

At Macy's, Colleen was busy working.

She was serving a customer who wanted to know where he could buy a necklace for his wife.

**D:** She replied: ''Ah, jewellery is actually on the second floor. Some items are at a discount.''

**I:** She replied that jewellery was actually on the second floor, adding that some items were at a discount.

**Q:** Did the customer wanted to buy his wife a necklace?

At the medical center, Henry was waiting to have an X-ray taken.

He fell off the bus this morning and feared he broke his leg, but worst of all, he was also very anxious about X-rays.

**D:** The doctor said: ''No need to worry! We will give you a lead board to protect you from radiation.''

**I:** The doctor told him not to worry because they would give him a lead board to protect him from radiation.

Julie's son had always been a bit of a couch potato.

Over dinner, Julie was talking about something she read in the newspaper to her husband.

**D:** She said: ''There, just read this article about child education! It exactly confirms my concerns about watching too much TV''.

**I:** She asked him to read the article about child education, for it exactly confirmed her concerns about watching too much TV.

**Q:** Was Julie worried her husband watched too much TV?

Filler items

A journalist was interviewing Eric, the older brother of a famous pop diva.

Eric did not really enjoy the attention he was given and wasn’t sure what to say.

**D:** He proclaimed: “I actually don’t see my sister very often these days. She is completely devoted to her career.”

At Mr. Harris's house, police officers Paul and Ken had some sad news to impart.

Mrs. Harris's husband, a well-known businessman, was killed in a car crash earlier this morning.

**D:** When Paul spoke with her, he said: “Mrs. Harris, I am afraid that your husband was involved in a road accident. He was pronounced dead at the scene.”

A medical emergency tutorial was taking place in the operating theater.

The tutor was demonstrating the steps to treat a heavy injury to the students.

**D:** He explained: “When the patient comes in, first set up the life-support machine and then stop the bleeding.”

**Q:** Is it correct to stop the bleeding first?

At the airport, Carol and Fraser were boarding the flight to Houston for a conference.

There were only five minutes left before the plane was taking off, and Carol started panicking because she couldn’t find her boarding card.

**D:** She turned to Fraser and said: “I can't find my stupid boarding pass! I must have left it at the duty-free shop!”

One of Melanie’s students, Jason, came into her office and said he could not reach her this morning.

Melanie was confused because she had been in her office the whole time.

**D:** She said: “Well, in that case, there must be something wrong with my telephone.”

**Q:** Did Jason try to phone Melanie in the morning?

It was February 14th. Carolyn and Tony were on a date at the newly opened Chinese restaurant.

The past couple of weeks, Carolyn was desperately trying to lose some weight.

**D:** So she said to Tony: “I hope you don’t mind if I’m just having a starter.”

At the party on Friday night, some new faces were to be seen.

Clare immediately caught Justin’s eye, who went over to her and offered her a drink.

**D:** Clare replied: “Thanks very much, but I don’t drink any alcohol actually.”

Britney is a student at the University of Minnesota.

After a heavy snow in the afternoon, she was complaining to her boyfriend James about the weather.

**D:** She said: “I really hate the winter! It’s always dark and the roads are too slippery.”

A famous symphony was going to be played for free at the university concert hall.

Kate and Andrew were reading the ad, and Kate looked particularly interested.

**D:** She said: “It sounds really good and it’s for free! I’m definitely going to that concert.”

**Q:** Was Kate excited about the symphony?

Two college students were talking about online shopping.

Fraser thought it was pointless and boring, but Brenda couldn't disagree more.

**D:** She said: ''Online shopping is absolutely amazing! I just snapped up some really cheap DVDs on Amazon.”

**Q:** Did Fraser find online shopping boring?

It was 6-year-old Herbert’s first day at school.

His mother Laura, who had never let him play with other children before, was very anxious.

**D:** She said to her husband: “I’m really not sure whether Herbert is ready for school. I'd prefer to wait for another year.”

Theatrical agent Peter was visited by one of his clients.

Shauna, a talented but struggling actress and singer, was desperate for a part in the new musical.

**D:** She said: “It’s embarrassing to say, but without this job, I won't even be able to pay for electricity.”

Maureen had helped David a lot with his coursework, so David wanted to invite her for dinner.

Asked what kind of food she would fancy, Maureen first thought for a second.

**I:** Then she replied that although she would normally not be that adventurous, perhaps she should try some sushi.

Jane was a new post-graduate at the department.

When she signed up at the Sports Club, she was having a conversation with Gregg about her favorite sports.

**I:** She said that although she had not practised a lot recently, she used to be quite good at tennis.

Leon and Betty were trying to get a mortgage for a new flat.

While Betty was studying the relevant newspaper ads, Leon was browsing some websites.

**I:** After a while, he said to Betty that it looked as if nobody would give them an instant cashback.

**Q:** Was Betty browsing the websites?

Harry and Leona were enjoying an afternoon walk in the park.

When they were approaching the pond, Harry was impressed by this peaceful place.

**I:** He found that it was so relaxing after a busy week, and that it was almost magical.

After making a small fortune in the lottery, Pamela bought quite an expensive car.

Today in the car park, her colleague Ralph was extremely impressed by her new possession.

**I:** He asked her whether that was her new car, adding that it looked really stylish.

**Q:** Was Pamela impressed by Ralph’s new car?

Daniel was injured in a car crash last week. At the hospital, he was quickly recovering.

Today, he was surprised that some distant relatives came to visit him.

**I:** Not without sarcasm, Daniel said that it looked as if one had to get hurt to get some flowers.

**Q:** Was Daniel involved in a car accident?

PhD student Ella was summoned to her supervisor Jim’s office to give a report on her current progress.

When Ella asked for an extension, Jim looked concerned.

**I:** He said that they really needed those data in by next month for that conference.

**Q:** Was Ella Jim’s PhD student?

Albert was taking Joanne to the new Schwarzenegger movie.

Albert was very excited, but since Joanne seemed not very keen, he tried to persuade her to go with him.

**I:** He said he was sure she would love it, and that the movie had some really cool special effects.

**Q:** Did Joanne want to see a Schwarzenegger movie?

Neil and Stephanie were visiting the local brewery.

Neil was amazed by the range of different beers produced, and he tried an American ale.

**I:** He said it was really strong and that he was glad he didn’t have to drive them home.

School teachers Duncan and Liana were arguing about the curriculum.

Apparently, Duncan was not a big fan of Liana’s modern style of teaching.

**I:** He said that if his own children were at this school, he’d make sure they would never have to attend her lessons.

**Q:** Did Duncan dislike Liana's way of teaching?

It was early Tuesday morning when Claire was looking for the train tickets she left in one of her handbags the other day.

After searching for a couple of minutes she became worried and confused.

**I:** She said to her partner Gareth that it was weird because she was pretty sure that they were in that black leather bag, but now they were gone.

**Q:** Was Claire looking for her bus tickets?

The heat of the morning was intense but Charles and Lucy had managed to climb up a hill.

From the top, Charles could see right down to the beach on the other side.

**I:** Excited, he suggested to Lucy that they should run down to the beach and take a plunge into the sea.

**Q:** Did Lucy suggest that they should take a plunge into the sea?

* please note that we used only half of the comprehension questions that are displayed in this Appendix in Experiments 1a and 1b
