# Supplementary material for: The Influence of Direct and Indirect Speech on Mental Representations
Source: PLoS One. 2013 Jun 12;8(6):e65480. doi: 10.1371/journal.pone.0065480 (PMC3680483; doi:10.1371/journal.pone.0065480)
Supplement: Appendix S3 — Materials that were used in Experiments 2 and 3. (DOC) [file pone.0065480.s003.doc]

Appendix S3 - Materials that were used in Experiments 2** and 3 (**D** = direct speech quotation, **I** = indirect speech quotation, **Q** = comprehension question, probes are underlined).

Experimental items

**D:** John walked into the room and said energetically, “I finally found my car keys.”

**I:** John walked into the room and said energetically that he finally found his car keys.

His wife was relieved because now they would make it in time for the concert.

**D:** The waiter came up to the table and declared hurriedly, “I’ll be right back to take your order.”

**I:** The waiter came up to the table and declared hurriedly that he would be back for their order.

Both friends used this extra time to take a look at the menu again.

**D:** The young woman sprinted onto the bus and asked frantically, “Do you go to the train station?”

**I:** The young woman sprinted onto the bus and asked frantically if it was going to the train station.

Because the bus would make a stop at the train station the woman got on the bus.

**Q:** Did the young woman ask whether the bus was going to the train station?

**D:** Macy skimmed over her recipe and said disappointedly, “I need to go grocery shopping.”

**I:** Macy skimmed over her recipe and said disappointedly that she needed to go grocery shopping.

She grabbed her coat and her car keys and went on her way.

**D:** Dan walked into the office and asked urgently, “Has anybody seen my cell phone?”

**I:** Dan walked into the office and asked urgently if anybody had seen his cell phone.

No one seemed to respond so Dan decided to call his mobile.

**Q:** Does Dan want to know whether someone had seen his car keys?

**D:** Andy strolled up to his mom and announced casually, “I just got accepted to Harvard!”

**I:** Andy strolled up to his mom and announced casually that he just got accepted to Harvard.

His mom was so happy that she made a reservation in a fancy restaurant to celebrate this good news.

**D:** The professor walked up to the cheating student and remarked swiftly, “You need to leave the exam.”

**I:** The professor walked up to the cheating student and remarked swiftly that he must leave the exam.

The student felt ashamed and left quietly.

**D:** Julie plodded through the room and announced lethargically, “I’m going to be late for work.”

**I:** Julie plodded through the room and announced lethargically that she was going to be late for work.

She didn’t feel like hurrying because she knew her boss wouldn’t come in before lunch.

**D:** The woman tentatively took her husband’s hand and whispered happily, “We’re having a baby.”

**I:** The woman tentatively took her husband’s hand and whispered happily that they were going to have a baby.

The husband was speechless and gave his wife a big hug.

**D:** The doctor went into the patient’s room and declared sadly, “Your tests did not come back normal.”

**I:** The doctor went into the patient’s room and declared sadly that the tests did not come back normal.

The patient worriedly looked at the doctor.

**D:** The dad wandered into his son’s bedroom and announced listlessly, “It’s time to get up.”

**I:** The dad wandered into his son’s bedroom and announced listlessly that it was time to get up.

Unexpectedly, the son was already quite awake.

**D:** The student walked up to the teacher and said regretfully, “I can’t make it to class this Friday.”

**I:** The student walked up to the teacher and said regretfully that she could not come to class Friday.

The teacher was okay with but decided to give the student another assignment.

**Q:** Will the student be able to make it to class on Friday?

**D:** Joe’s boss darted into his cubicle and declared excitedly, “You gave a great presentation.”

**I:** Joe’s boss darted into his cubicle and declared excitedly that he gave a great presentation.

Joe was very happy about this comment and hoped for a promotion.

**D:** Ellen walked out of the dressing room and asked carefully, “Does this dress look good on me?”

**I:** Ellen walked out of the dressing room and asked carefully if the dress looked good on her.

Because her best friend loved the dress Ellen decided to buy it.

**D:** The stylist held up a mirror and asked expectantly, “Do you like your new style?”

**I:** The stylist held up a mirror and asked expectantly if she liked her new style.

Unfortunately, the customer thought her new haircut didn’t look good on her.

**Q:** Did the stylist hold up a mirror?

**D:** The lawyer stared at the jury and asserted certainly, “I know my client is innocent.”

**I:** The lawyer stared at the jury and asserted certainly that he knew his client was innocent.

After this statement, the jury withdraw for consideration.

**D:** The coach ran onto the court and yelled clearly, “You have to make your free throws!”

**I:** The coach ran onto the court and yelled clearly that they had to make their free throws.

In the end the coach was satisfied because his team won.

**D:** The reporter dashed up to the politician and inquired hastily, “Are you running for mayor?”

**I:** The reporter dashed up to the politician and inquired hastily if she was running for mayor.

The politician however didn’t want to comment on that.

**D:** Amy walked up to the store manager and asked immediately, “Where is the shoe department?”

**I:** Amy walked up to the store manager and asked immediately where the shoe department was.

Half an hour later she found exactly the boots that she was looking for.

**Q:** Was Amy looking for the shoe department?

**D:** Nick glanced at his watch and said briskly, “The movie starts in five minutes.”

**I:** Nick glanced at his watch and said briskly that the movie started in five minutes.

He made his girlfriend to hurry up to get to the cinema in time.

**D:** Leslie went into the gym and said softly, “I’m nervous for my first yoga class.”

**I:** Leslie went into the gym and said softly that she was nervous for her first yoga class.

Because of that the yoga instructor started his lesson by introducing everyone.

**D:** Jack got into his car and muttered unexcitedly, “I need to get gas today.”

**I:** Jack got into his car and muttered unexcitedly that he needed to get gas today.

He did so later that day on his way back home.

**Q:** Did Jack need to get Advil today?

**D:** Andrea pulled into the parking lot and yelled irritatedly, “Somebody parked in my space again!”

**I:** Andrea pulled into the parking lot and yelled irritatedly that somebody had parked in her space again.

It was hard to find another space but in the end Andrea succeeded.

**D:** The cab driver turned around and asked tiredly, “Where do you need to go?”

**I:** The cab driver turned around and asked tiredly where they needed to go.

Because the tourists didn’t know where to go to the cab driver dropped them of at the tourist information.

Filler items

**D:** The bartender approached the woman and asked seductively, “What can I get you?”

The woman was not impressed and ordered her favorite cocktail.

**D:** After the manager found out about the mistake, Mike said defensively, “That has nothing to do with me.”

The manager believed him and wanted to know who was responsible.

**D:** Maria walked up to the cashier and asked anxiously, “Did you find my wallet?”

The cashier smiled friendly and handed Maria her wallet.

**Q:** Is Maria looking for her wallet?

**D:** Peter walked up to his colleagues and yelled hysterically, “There’s a fire alarm. We have to leave the building now!”

When they stood outside the building they saw a lot of smoking coming out of it.

**D:** Jim walked up to his girlfriend and said enthusiastically, “I booked us tickets to the Caribbean”

She was so excited about this that she could hardly wait to pack her suitcase.

**Q:** Did Jim book his friend a ticket to the Caribbean?

**D:** The teenager saw his mother going to the kitchen and said lazily, “Can you get me another soda?”

Because she didn’t want to disappoint him she poured him another glass.

**D:** Katie opened the front door and said hesitantly, “What are you doing here?”

At the door stood her sister who she hadn’t seen for a while because of an argument.

**D:** The psychiatrist looked at his patient and said calmly, “I don’t think you are ready to go home.”

The patient looked depressed when he realized he had to stay for another weekend.

**D:** The officer arrested the suspect and said sarcastically, “We are going for a nice little ride.”

The suspect tried to convince the officer of his innocence but he wasn’t successful.

**D:** The rocker approached the audience and yelled loudly, “Let’s have a party!”

The audience felt like it and in the end everyone thought the concert was a big success.

**Q:** Was the audience attending a rock concert?

**D:** The mother carried her baby and said worriedly, “What can I do to make you stop crying?”

At the end of the day, she decided to call her mum and ask her for advice.

**D:** The young boy stayed in the car and yelled defiantly, “I don’t want to go to the dentist.”

His father got really angry, grabbed his son’s arm and forced him to come with him.

**I:** Martin noticed that his ex girlfriend had gained weight so he asked deliberately whether she was pregnant.

She got upset by his question and walked away without saying another word.

**I:** The museum guard walked around and announced repeatedly that people had to be quiet.

He wondered why people couldn’t just watch the paintings without talking.

**I:** The service employee at the train station announced frequently that the train to New York would be delayed.

Twenty minutes later the train to New York finally entered the train station.

**I:** George got home to his wife who did not feel well so he said thoughtfully that he brought her some food.

She thought it was really sweet of him to get her something but she didn’t feel like eating.

**I:** The student walked up to his parents and said promptly that his laptop was broken.

His parents thought their son really needed to learn to be more careful with his belongings.

**Q:** Did the student break his bag?

**I:** In the theater, Josh looked at the man next to him and told him rudely that he had to be quiet.

The man apologized and stayed quiet until the end of the movie.

**I:** At a very nice dinner, Mark stood up and asked respectfully whether he could be excused for a moment.

After a quick call from his boss Mark came back and enjoyed the rest of the dinner.

**Q:** Did John stand up and ask to be excused for a moment?

**I:** The manager looked at his watch and said quickly that he had to go.

He had planned a very important lunch meeting.

**I:** The teenager called her mother and remarked nonchalantly that she wouldn’t be home until midnight.

The mother sighed deeply as the teenager hang up the phone.

**I:** The nurse sat down with the patient and said reassuringly that the surgery wouldn’t take that long.

After that she anesthetized the patient and brought him to the operating room.

**I:** The young woman sang to the jury and asked insistently whether they liked her performance.

Unfortunately, the jury wasn’t impressed so the woman didn’t make it to the next round.

**I:** Martha ran upstairs to her husband and announced abruptly that they had to get going.

They were running late and Martha wanted to arrive at their daughters birthday in time.

**Q:** Did Martha and her husband need to leave?

** please note that we used only the direct or indirect speech sentence in Experiments 2a and 2b
